# Capsaicin Potently Blocks *Salmonella typhimurium* Invasion of Vero Cells

**DOI:** 10.3390/antibiotics11050666

**Published:** 2022-05-16

**Authors:** Joseph A. Ayariga, Daniel A. Abugri, Balagopal Amrutha, Robert Villafane

**Affiliations:** 1 The Biomedical Engineering Program, College of Science, Technology, Engineering and Mathematics (C-STEM), Alabama State University, Montgomery, AL 36104, USA; 2 Department of Biological Sciences, College of Science, Technology, Engineering and Mathematics (C-STEM), Alabama State University, Montgomery, AL 36104, USA; rvillafane@alasu.edu; 3 Microbiology PhD Program, College of Science, Technology, Engineering and Mathematics (C-STEM), Alabama State University, Montgomery, AL 36104, USA; 4 Laboratory of Ethnomedicine, Parasitology, and Drug Discovery, College of Science, Technology, Engineering and Mathematics (C-STEM), Alabama State University, Montgomery, AL 36104, USA; 5 Division of Oncology, College of Medicine, University of Saskatchewan, Saskatoon, SK S7N 5E5, Canada; amrutakikka@gmail.com

**Keywords:** *Capsicum chinense*, capsaicin, inhibition, *Salmonella typhimurium*, growth

## Abstract

*Salmonella typhimurium* (*S. typhimurium*) is one of the major food and waterborne bacteria that causes several health outbreaks in the world. Although there are few antibiotics against this bacterium, some of these drugs are challenged with resistance and toxicity. To mitigate this challenge, our group explored the ethnomedicinal/herbalism knowledge about a certain spice used in Northern Ghana in West Africa against bacterial and viral infection. This plant is *Capsicum chinense* (*C. chinense*). The plant is one of the commonest food spices consumed across the world. The seed of the plant contains both capsaicin and dihydrocapsaicin. Apart from capsaicin and dihydrocapsaicin, other major capsaicinoids in *C. chinense* include nordihydrocapsaicin, homodihydrocapsaicin, and homocapsaicin. In this pilot work, we investigated the antibacterial activity of pure capsaicin and capsaicin extract obtained from *C. chinense* against *S. typhimurium* in vitro. Capsaicin extract showed potent inhibition of *S. typhimurium* growth at concentrations as low as 100 ng/mL, whereas pure capsaicin comparatively showed poorer inhibition of bacteria growth at such a concentration. Interestingly, both capsaicin extract and pure capsaicin were found to potently block a *S. typhimurium* invasion of the Vero cell in vitro. Taken together, we believed that capsaicin might work synergistically with dihydrocapsaicin or the other capsaicinoids to inhibit *S. typhimurium* growth, whereas individually, capsaicin or dihydrocapsaicin could potently block the bacteria entry and invasion of Vero cells.

## 1. Introduction

Over the last 4 to 5 decades, the infections related to nontyphoidal *Salmonella* have increased and continues to be a major global burden in health care systems in most countries [1,2,3,4,5]. Most outbreaks of *S. typhimurium* are facilitated through the acquisition of new traits that enhance their adaptability and virulence [6,7]. For instance, the emergence of multidrug-resistant (MDR) *S. typhimurium* DT104 has been demonstrated to be caused by the acquisition of the MDR gene via a plasmid-mediated process [8]. For example, the MDR-AmpC phenotypes *S. typhimurium* and *S. newport* have been demonstrated to be exclusively plasmid mediated by plasmid transfer MDR genes [9].

*S. typhimurium* is a Gram-negative bacterium that causes gastroenteritis, bacteremia, and focal infections [4,7]. The symptoms include high fever and diarrhea [1,4,5,6]. The infection of *S. typhimurium* is currently treated with trimethoprim and sulfamethoxazole combination, or with ciprofloxacin, azithromycin, or ceftriaxone [9]. However, the potential to develop resistance against these drugs are high. Most nontyphoidal *Salmonella* causes mild to severe clinical symptoms and could be life-threatening to individuals with weak immune systems [10,11]. In the early 2000s, about 600 deaths were recorded per year in the United States alone due to infections with nontyphoidal *Salmonella* serotypes [12]. The resistance of Salmonellae to antimicrobials has been demonstrated to correlate with an increased risk of hospitalization, invasive illness, and death [13,14,15,16,17,18,19].

Several studies have documented the importance of natural bioactive compounds’ antimicrobial properties [20,21,22]. Capsaicin, a naturally existing bioactive compound found in *C. chinense*, has received extensive scientific interest because of its pharmacological and biological properties and its mechanism of action against pathogen growth and survival [23,24,25,26,27,28]. Specifically, capsaicin has been shown to exert anti-inflammatory and pain relieving [29], anti-cancer, anti-tumor [30,31], anti-cardiovascular, and gastrointestinal effects [32,33]. Dietary capsaicin has been demonstrated to protect cardiometabolic organs from dysfunction [32]. Studies underlying the pharmacodynamics of capsaicin revealed that peroxidase is directly linked to capsaicin metabolism, for which peroxidase oxidizes capsaicin [34]. Peroxisomes are cellular compartments where the turnover of some reactive anionic species and complex lipids take place. In the peroxisomal compartment, capsaicin could be degraded by the peroxisomal β-oxidation enzymes into acyl-CoAs which in turn served as a substrate for the mitochondrial carnitine [35].

In a study on the antimicrobial property of capsaicin, Manini et al., (2015), demonstrated that *Salmonella enteritis* (*S. enteritis*) did not develop any resistance against capsaicin treatment, and they showed that sub-lethal concentrations of capsaicin blocked *S. enteritis* from adhering to A549 monolayers cells, and significantly reduced cell-invasiveness and hemolytic activity [28].

In another study, Omolo et al., (2018) showed that capsaicin from the fruits of *C. chinense* cultivars demonstrated bactericidal and antifungal effects [27]. They revealed that *L. monocytogenes* and *S. aureus* were more susceptible to capsaicin than *Salmonella* and *E. coli* O157:H7, and that *C. albicans* was the most susceptible to capsaicin [27]. Qiu et al., (2012) revealed that capsaicin provided protection for mice from methicillin-resistant *Staphylococcus aureus* infection [36]. Chatterjee et al., (2010) also demonstrated that capsaicin is a potential inhibitor of cholera toxin production in *Vibrio cholerae* [37].

Capsaicin continues to attract major scientific interest specifically relating to its inhibitory potential against foodborne pathogens [38], *Helicobacter pylori* [39] and *Pseudomonas aeruginosa*, against MDR-ESBL, producing *Escherichia coli* [40].

The development of antimicrobial resistance is frequent with *S. typhimurium*. For instance, *S. typhimurium* DT104 has been shown to be resistant to cephalosporins, trimethoprim, ampicillin, chloramphenicol, quinolones, streptomycin, sulfonamides, and tetracycline [41]. Although the global survey of Salmonellosis has been documented [42], relatively little is known about the interaction of the causative organism of Salmonellosis (which is *Salmonella,* enteric bacterium) and capsaicin (one of the bioactive compounds commonly consumed as food spice). This study was carried out to assess the effect of capsaicin and capsaicin extract on the growth of *S. typhimurium* in vitro and their mechanism of action.

## 2. Results and Discussion

### 2.1. Effect of C. chinense Extract and Pure Capsaicin on S. typhimurium Growth

**Figure 1:**
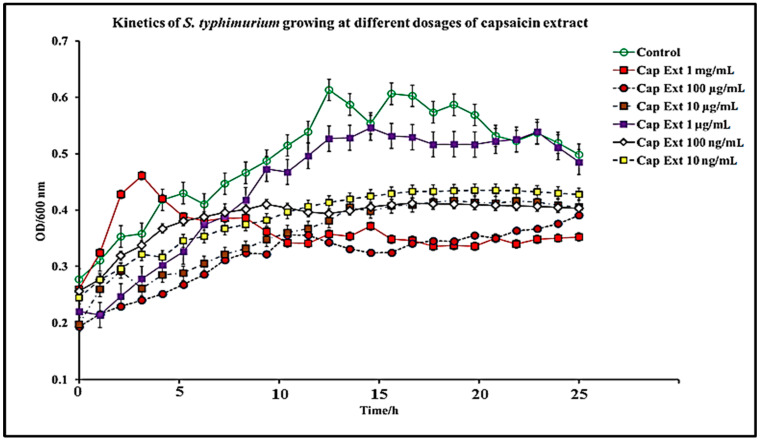
Kinetic dose and time-dependent effect of capsaicin extract on *S. typhimurium* growth in vitro. The data are represented as means of three independent experiments ± standard deviation.

**Figure 2:**
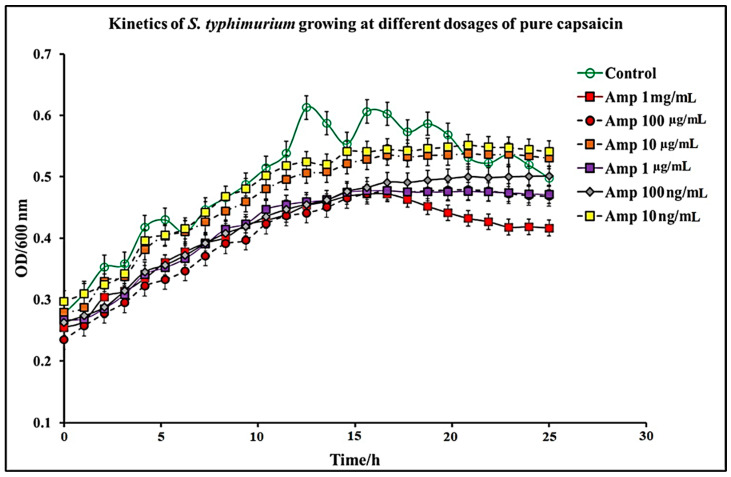
Kinetics of *S. typhimurium* growth at varying dosages of pure capsaicin. The results represent the average of three independent experiments ± standard deviation.

**Figure 3:**
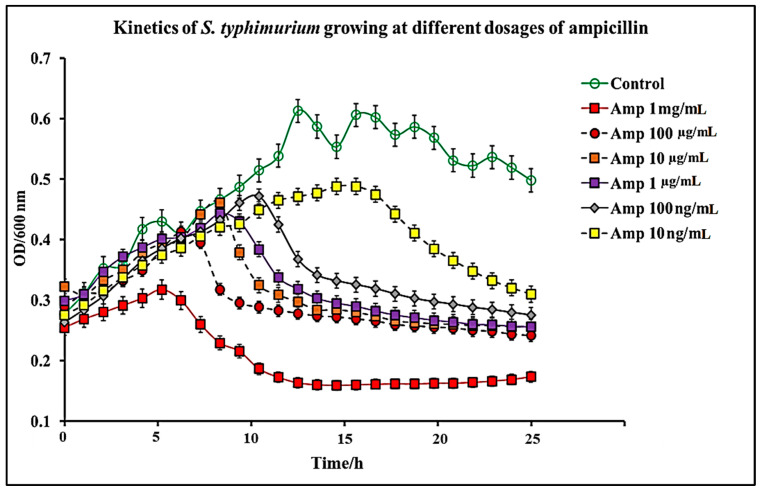
Kinetics of *S. typhimurium* growth at varying dosages of ampicillin. The results represent a mean of three independent experiments ± standard deviation.

**Figure 4:**
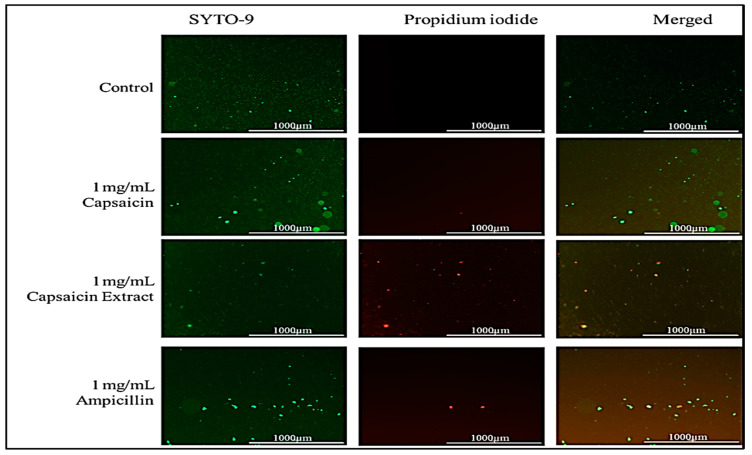
Immunofluorescent images of *S. typhimurium* growing on culture media pretreated with 1 mg/mL of pure capsaicin or capsaicin extract or ampicillin for 30 min incubation. Control received no treatment. Undamaged bacterial membranes are shown via green fluorescence, but those with damaged membranes are indicated via red fluorescence.

**Figure 5:**
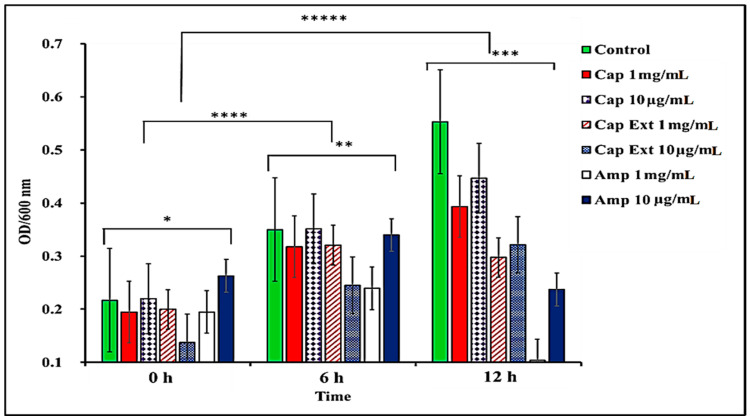
Comparison of pure capsaicin, capsaicin extract, and ampicillin ability to inhibit *S. typhimurium* growth at various culturing time points (0, 6, and 12 h) interactions. * and ** *p*-value ≤ 0.1, ***, ****, and ***** *p*-value ≤ 0.05; *n* = 3.

In this study, we report that pure capsaicin or capsaicin extract isolated from *C. chinense* fruit exerted inhibitory activity against *S. typhimurium* growth in vitro. Similar reports of capsaicin and dihydrocapsaicin showing antibacterial properties have been published [28,43]. The capsaicin extract, however, exerted relatively higher inhibitory activity against *S. typhimurium* than the pure capsaicin at similar concentrations (Figure 1 and Figure 2). The lowest concentration of capsaicin extract that potently reduced *S. typhimurium* growth was 10 ng/mL, whereas pure capsaicin could not effectively reduce the growth of the bacteria at concentrations of 10 µg/mL or higher (Figure 2). In a control experiment using ampicillin at varying concentrations, a lower concentration of 10 ng/mL potently reduced the growth of *S. typhimurium* (Figure 3). As shown in Figure 4, staining of *S. typhimurium* with SYTO-9 and propidium iodide (PI) indicated that *S. typhimurium* growing on culture media pretreated with 1 mg/mL of capsaicin extract showed higher *S. typhimurium* killing (indicated by the higher red fluorescence of the PI). The control group that received no treatment showed no red fluorescence (Figure 4) since the undamaged bacterial membrane showed green fluorescence, but those with damaged membranes shows red fluorescence. Immunofluorescent images of 100, 10, and 1 µg/mL concentrations of capsaicin, and capsaicin extract treatment of the bacteria are presented in the Supplementary Materials (Figures S1–S3). Similarly, as in Figure 2 and Figure 5 also showed that the capsaicin extract produced relatively higher inhibitory activity against *S. typhimurium* than the pure capsaicin at similar concentrations.

### 2.2. Cytotoxicity of Capsaicin

**Figure 6:**
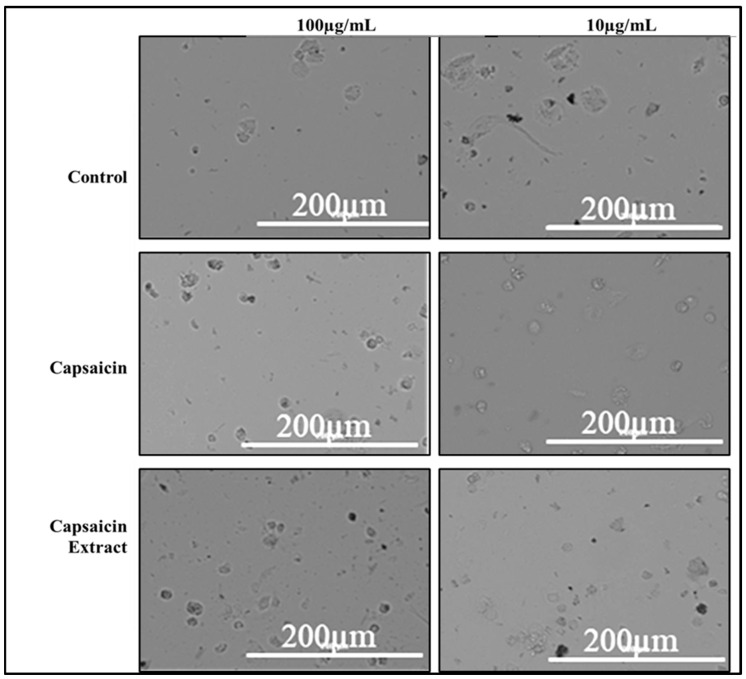
Phase contrast microscopy depicting Vero cells growing in media supplemented with capsaicin or capsaicin extract for 8 h interaction. Control group received no treatment.

**Figure 7:**
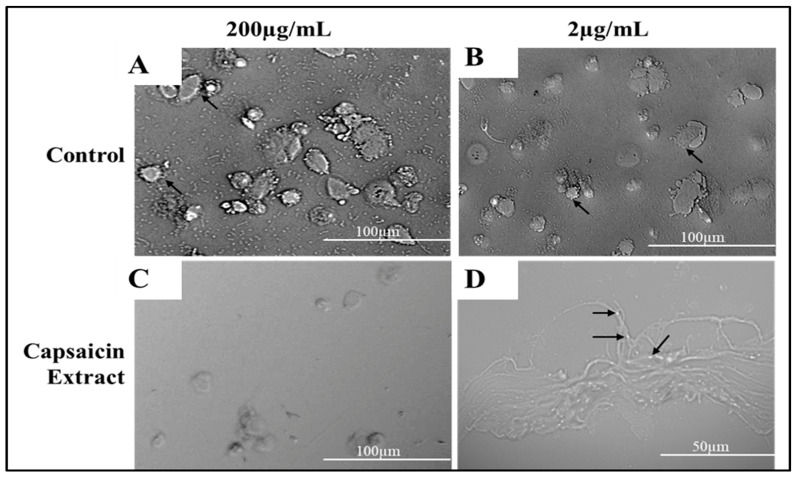
Phase contrast microscopy depicting Vero cells infected with *S. typhimurium* as control (**A**), and treated with capsaicin extract at 2 or 200 µg/mL (**B**,**C**), respectively. Experiments were treated for 8 h. Black arrows in (**B**) show attached *S. typhimurium* to Vero cells, (**C**) shows internalized *S. typhimurium* in Vero cells and (**D**) shows bacteria attached to Vero cell at higher magnification (50 µm) as indicated with black arrows.

Vero cells were cultured in DMEM medium supplemented with varying concentrations of pure capsaicin or capsaicin extract. The viabilities of these cells were shown not to be significantly different (*p* ≤ 0.05). However, we observed slightly higher growth of Vero cells at 0.1 µg/mL of capsaicin than all other treatment groups (Figure S4). Morphologically, no distinct differences were observed between cells of the different treatments’ groups of capsaicin, or capsaicin extract (Figure 6). Infecting Vero cells with *S. typhimurium* showed that the bacteria attached to host Vero cells within 30 min post-infection (Figure 7).

### 2.3. Adherence of S. typhimurium to Vero Cells

The intracellular presence of *S. typhimurium* can constitute a major reservoir of continuing bacteria in vivo that can subsequently cause reinfections. The survival of *S. typhimurium* inside the host cell requires first the bacteria’s ability to attach to the host, penetrate the host cell, and invade it. Thus, the invasion and survival of *S. typhimurium* in the Vero cells present a huge challenge to drugs that do not act intracellularly, e.g., penicillin.

**Figure 8:**
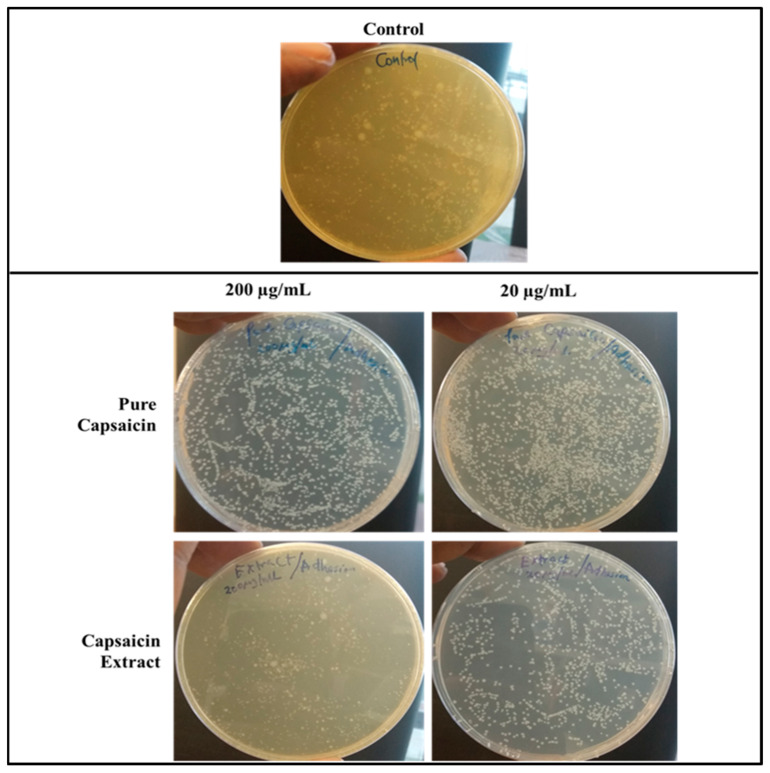
Images of Agar plates depicting the CFUs of *S. typhimurium* in bacteria adhesion assay. Monolayer Vero cells grown in media supplemented with capsaicin at varying concentrations were infected with *S. typhimurium* and incubated for 30 min, washed thrice with 1X PBS to remove bacterial cells in suspension. Vero cells were then lysed with chilled distill water and plated on Agar plates overnight. The treated samples received capsaicin extract at 200 or 20 µg/mL respectively, whereas the control received 1X PBS.

Using the anti-adherence assay, we demonstrated both pictorially and quantitatively the adhesion of *S. typhimurium* to Vero cells after 30 min post infection. Vero cells were inoculated with *S. typhimurium* for 30 min as described in anti-adhesion assay in materials and methods. The capsaicin extract treated group showed very low bacteria adhesion (Figure 7 and Figure 8) as compared to the control and the pure capsaicin treated samples. Quantitatively, at *p*-values below 0.05, there was no significant difference between the CFUs counted between the different groups, indicating that adherence to cells were not significantly affected by treatment to pure capsaicin or capsaicin extract (Figure S5A). The remarkably high number of bacteria adhering to Vero cells at pure capsaicin concentration of 200 µg/mL was contrasted sharply with the drastic decrease in the number of intracellular bacteria that were record in presence of same concentration of pure capsaicin concentrations (Figure 8 and Figure S5B). There were slightly lower number of bacteria adhering to Vero cells when treated with 200 µg/mL of capsaicin extract (Figure 8).

### 2.4. Invasion of S. typhimurium into Vero Cells

**Figure 9:**
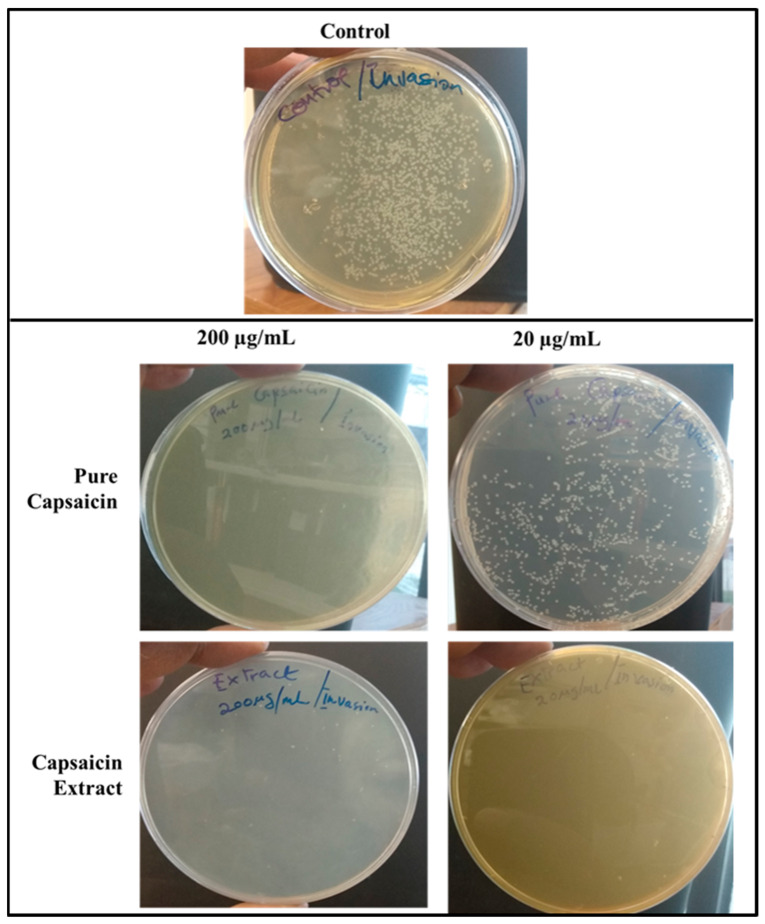
Images of Agar plates depicting the CFUs of *S. typhimurium* in bacteria invasion assay. Monolayer Vero cells grown in media supplemented with capsaicin at varying concentrations were infected with *S. typhimurium* and incubated for 2 h to allow for bacterial cell invasion of Vero cells. Then the infected monolayer Vero cells were washed thrice with 1X PBS to remove bacterial cells in suspension and followed with antibiotic treatment for 2 h at 37 °C in 5% CO_2_ to kill bacterial cells that adhered to Vero cells but did not internalize. Then antibiotics were washed off and Vero cells lysed with chilled distill water and plated on Agar plates overnight. The treated samples received capsaicin extract at 200 or 20 µg/mL, respectively, whereas the control received 1X PBS.

The internalization of *S. typhimurium* into the host cells is indicative of invasion. To investigate the capacity of pure capsaicin or capsaicin extract in blocking the entry of *S. typhimurium* into Vero cells were carried out (Figure 9 and Figure S5B). It was evident that at 200 µg/mL both pure capsaicin and capsaicin extract potently blocked the invasion of *S. typhimurium* into Vero cells since very few CFUs could be observed (Figure 9) or recorded (Figure S5B).

### 2.5. Effect of Capsaicin or Capsaicin Extract on S. typhimurium Membrane Integrity

To evaluate the mechanism of action of capsaicin or capsaicin extract on *S. typhimurium*, we tested if the compounds acted by disrupting bacterial cell membrane structure which might lead to cell membrane lysis. Lysed bacterial cells will have their genetic content extracellularly and will be subjected to migrate through the matrix of the gel. However, intact cell membranes will keep DNA inside cells and block their movement through the matrix of the gel. To test this hypothesis, 100 µL of *S. typhimurium* cells grown to OD of 0.5 was pelleted by centrifugation and re-suspended in 1X PBS, followed by incubation with lysozyme, capsaicin, capsaicin extract, or ampicillin at concentrations 100 or 10 µg/mL for 20 or 60 min. After the set time points of incubations, 50 µL of each treatment was mixed with Agarose gel loading dye (Trackit, Thermo Fisher Scientific, Waltham, MA, USA). Samples were run in 0.8% agarose gel and gel stained with Ethidium bromide for 30 min.

As depicted in Figure S6, *S. typhimurium* membrane integrity assessment via agarose gel electrophoresis was carried out to ascertain the effect of pure capsaicin or capsaicin extract on the *S. typhimurium* membrane integrity. As shown in Figure S6A, the bacterial cells incubated for a brief period (20 min) showed no band intensities nor band migrations except for lane capsaicin extract lane 100 µg/mL. In addition, in Figure S6B, bacterial cells were incubated for 60 min which showed some significantly higher band intensities in all wells. Trails of blurry band was observed in Figure S6B as shown by the black arrows at capsaicin extract lane 100 µg/mL. A fainter band can be observed at the 10 µg/mL lane too of capsaicin extract. As can be observed in Figure S6A, no blurry band trail could be seen. However, we observe a band at the well for 100 µg/mL capsaicin extract, with no bands observed in all other wells. This might indicate that at 20 min treatment, most bacterial cell membrane at all treatment groups except the 100 µg/mL capsaicin extract treatment group still maintained their cell membrane integrity. However, with continued incubation for 60 min, the compounds created slight changes to the membrane structures of the bacteria, allowing for the membrane impermeable dye to enter the bacteria and intercalate with the bacterial DNA, hence giving off significantly high band intensities.

## 3. Discussion

In this work, we screened the effect of pure capsaicin and capsaicin extract against *S. typhimurium*, one of the major food- and water-borne bacteria that causes several health outbreaks in the world.

*S. typhimurium* is a Gram-negative bacteria composed of two membranous structures: The inner one known as the plasma membrane which is made of a phospholipid’s bilayer, and the outer membrane which consists of proteins, including porins, receptors, and an asymmetric distribution of lipids [44]. The effect of pure capsaicin or capsaicin extract from *C. chinense* on this bacterium has been assessed via several assays. We proved that pure capsaicin and capsaicin extract showed potent antibacterial activity against *S. typhimurium*; however, the capsaicin extract performed better at inhibiting the bacterial growth than the pure sample. At higher concentrations of the capsaicin extract, the reduction in bacterial cell growth was comparable to the well-known antibiotic ampicillin (Figure 1, Figure 3 and Figure 5).

The antibacterial property of the capsaicin extract or the pure capsaicin against *S. typhimurium* was also assessed via live/dead assay using SYBR green I or SYTO-9 and propidium iodide, in which bacteria with intact cell membranes gives off green fluorescence, whereas cells with damaged membranes show red fluorescence. Our assay showed that capsaicin extract exhibited higher bactericidal activity against *S. typhimurium* than the pure compound. This we presumed might be due to membrane disruption as proposed by the higher red fluorescence. This inference has also been proposed by early researchers [28,45,46,47].

Both pure capsaicin and capsaicin extract at lower concentrations showed minimal to no effect on Vero cell growth inhibition. On the contrary, below 1 µg/mL, both the pure capsaicin and capsaicin extracted seemed to promote Vero cell growth.

To examine the mechanism of action of the pure capsaicin or capsaicin extract on *S. typhimurium* killing, we postulated bacterial membrane damage. To test for this hypothesis, we employed ethidium monoazide which is a fluorescent dye that binds covalently to nucleic acids however it is cell membrane-impermeable. To therefore bind to cell’s nucleic acid then such bacteria must have compromised membranes [48]. Our assay revealed that a few minutes of treatment of capsaicin extract to *S. typhimurium* was sufficient to significantly damage the bacterial cell membrane hence allowing the entry of the dye into whole cells loaded into the lane (Figure S6A, lane 100 µg/mL of capsaicin extract). In Figure S6B, however, when the bacterial cells were incubated for a longer time point, significantly higher band intensities in all wells were recorded: nonetheless, no DNA migration through the matrix of the gel was recorded except for the capsaicin extract treatment groups. The leaking of genome out of the cells might be indicative of cell lysis, and not minor cell membrane structural damages, thus allowing the cytoplasmic content to flow extracellularly. As indicated in Figure S6B, only capsaicin extract treatment causes this leaking phenomenon at 1-h post-treatment. This seems to indicate that even though capsaicin does exert membrane damage, the pure capsaicin does not cause cell lysis; however, the capsaicin extract at longer incubation caused cell lysis.

This property of capsaicin’s mechanism of action as an antimicrobial is an interesting and useful discovery. Further studies are required at this stage to show the biochemical mechanism of action of capsaicin extract’s antibacterial activity against *S. typhimurium*. However, studies on the effect of capsaicin on biomimetic membranes provide a perfect starting point for consideration. Using a laurdan fluorescent molecular probe, Sharma et al., (2019) demonstrated the effect of capsaicin on membrane fluidity, which is a very crucial biophysical property of membranes [46]. They demonstrated that liposomes with higher capsaicin concentrations (10% and 20%) showed higher fluidity than lower capsaicin concentrations (capsaicin 5%). Additionally, this group demonstrated that the capsaicin caused thermo-induced membrane excess area, hence promoting liposomes to fluctuate more upon increasing temperature [46].

Since capsaicin and its derivatives are mostly lipophilic in nature, their interaction with cell membranes presents an important pharmacological and physiological phenomenon. Even though, it is a published fact that this molecule selectively binds to the transient receptor vanilloid 1 (TRPV1) [49] this specific mechanism might not be the case in this study since we are dealing with prokaryotic organisms. This molecule is an amphiphilic hydrophilic 4-hydroxy-3-methoxybenzyl-8-methylnon-6-enamide group and a hydrophobic 7-methyl-8-octene moiety [46]. For this reason, capsaicin has the capacity to insert itself into the plasma membrane phospholipid bilayer that could substantially certain biophysicochemical conditions cause “membrane structural discordance” or damage, leading to cell membrane permeability [50]. The lytic observation in the capsaicin extract treatment might be due to the synergistic effect of capsaicin and dihydrocapsaicin found in the extract instead of the pure capsaicin sample.

The observed capsaicin effect on *S. typhimurium* killing might also be due in part to other factors such as reactive oxygen production, membranes leaks that disrupts bacterial energy biosynthesis, and the intracellular influx of ions that can potentially disrupt cellular processes. Capsaicin has also been known to be involved in lipid oxidation [33], the oxidation of the bacterial membrane might have downstream biological ramifications that might cause the bacterial cell death.

At the present stage, further work is needed to fully elucidate the mechanism of capsaicin or capsaicin extract blockage of *S. typhimurium* invasion, as well as definitively prove the involvement of capsaicin in the plasma membrane of these Gram-negative bacteria. Currently, ongoing work is being carried out in our laboratory to understand the relationship between *S. typhimurium* biofilm formation reduction in the presence of capsaicin as well as the molecular mechanisms orchestrating such observation. Investigating the biotransformation of capsaicin in *S. typhimurium* will also be an exciting undertaking.

## 4. Materials and Methods

### 4.1. Reagents

All chemicals (Hexane, methanol –LC-MS (≥99.9%), water, and ethanol absolute proof (≥99.5%) were all high-performance liquid chromatography (HPLC) grade from Sigma Aldrich, MO, USA. Capsaicin and dihydrocapsaicin standards were obtained from Santa Cruz Biotechnology Inc., Dallas, TX, USA. Dulbecco’s Modified Eagle Medium (DMEM) and FBS were purchased from ATCC (Manassa, CO, USA). The concentrations of the capsaicin and dihydrocapsaicin standards were evaluated using a stock solution of 6 mg/mL capsaicin and a stock solution of 5 mg/mL dihydrocapsaicin. The standards were dissolved completely in a 10:1 hexane-ethanol solution [31].

### 4.2. Animal Cells and Bacterial Strain

Bacterial strains of *Salmonella typhimurium* (BV4012 strain) were obtained from Dr. Robert Villafane laboratory, Alabama State University, Montgomery, AL, USA). The bacteria strain was grown and maintained in LB broth or LB agar, under 37 °C. Vero cells (ATCC, CCL 81) were obtained from BEI Resources (Manassas, VA, USA). These cells were cultured and maintained in a T-25 cm^3^ flask using DMEM supplemented with 10% FBS and 1% penicillium-streptomycin-amphotericin, at 37 °C, 5% CO_2_.

### 4.3. Description of Plant and Collection of Plant Material

*C. chinense* fruits were ground to a thick, semi-solid paste which had the characteristic red color of the fruit. The resulting fine paste was mixed with hexane and extracted exhaustively. The elute was air-dried to obtain a powdered red extract [31].

Growth kinetics of *S. typhimurium* were performed in 96-well plates containing varying concentrations of pure capsaicin, capsaicin extract, or ampicillin. Concentrations used for capsaicin or ampicillin were 1 mg/mL, 100 µg/mL, 10 µg/mL, 1 µg/mL, 100 ng/mL, and 10 ng/mL for pure capsaicin, capsaicin extract, or ampicillin. Growth kinetics were carried out using a similar procedure previously published by Marini et al., (2015) [28], with slight modifications. In summary, 300 µL of *S. typhimurium* in LB medium (~10 × 10^6^ CFU/mL) was placed, then quickly followed by varying doses of capsaicin (pure) or capsaicin extract in microtiter plates. Afterward, the plates containing *S. typhimurium* and capsaicin, or capsaicin extract were incubated for 24 h at 37 °C and read at OD600 at 1 h intervals using a SpectraMax^®^ ABS Plus Microplate Reader. *S. typhimurium* grown in LB medium in the absence of capsaicin or capsaicin extract served as controls. All experiments were performed in triplicate.

#### 4.3.1. *S. typhimurium* Live/Dead Assay

To test the bactericidal property of capsaicin or capsaicin extract from *C. chinense*, we employed the live/dead assay by using SYBR Green I (Invitrogen, Waltham, MA, USA) and propidium iodide (Sigma–Aldrich, St. Louis, MO, USA) following a protocol previously published by Magi et al., (2015) that made use of the two nucleic acid dyes that differ in their ability to penetrate bacterial cells. In short, *S. typhimurium* was grown overnight, and the overnight culture diluted to an OD600 of 0.2 using LB broth supplemented with varying concentrations of capsaicin, capsaicin extract, or ampicillin. An untreated sample served as a control. Treated samples were incubated at 37 °C, 5% CO_2_ for 60 min. Afterwards, samples were stained with 1 × SYBR Green I or with SYTO-9 and 40 µg/mL propidium iodide, at room temperature for 25 min, covered with aluminum foil. Subsequently, cells were harvested on filters (Ø = 0.2 µm, Millipore, Burlington, MA, USA), and examined under an EVOS FLC microscope (Life Technologies).

#### 4.3.2. *S. typhimurium* Membrane Integrity Test

To evaluate the mechanism of action of capsaicin or capsaicin extract on *S. typhimurium*, we tested if the compounds acted by disrupting bacterial cell membrane which might lead to cell membrane lysis. To test this hypothesis, 100 µL of *S. typhimurium* cells grown to OD of 0.5 was pelleted by centrifugation and resuspended in 1X PBS, followed by incubation with lysozyme, capsaicin, capsaicin extract, or ampicillin at concentrations of 100 or 10 µg/mL for 20 or 60 min. After the set time points of incubations, 50 µL of each treatment were mixed with Agarose gel loading dye (Trackit, Thermo Fisher Scientific, Waltham, MA, USA). Samples were run in 0.8% agarose gel and gel stained with Ethidium bromide for 30 min. Following rinsing, the agarose gel’s bands were captured using ChemiDoc installed with Quantity One software (Quantity One version 4.6.9, Bio-Rad Laboratories, Inc., Hercules, CA, USA).

### 4.4. Vero Cell Viability

To investigate the effect of capsaicin on Vero cells, the cells were cultured in 96-tissue well plates containing DMEM media supplemented with 10% FBS. Capsaicin at 1000 µg/mL, 100 µg/mL, 10 µg/mL, 1 µg/mL, 0.1 µg/mL, 0.01 µg/mL and 0.001 µg/mL was mixed with the culture media and incubated with capsaicin for 8 h. Control cultures did not receive capsaicin treatment. At the 8 h time point, AlamarBlue at 10% was added to the growing culture and allowed to incubate for an additional 4 h. Absorbance at 570 nm was read using Cytation 3 imaging/plate reader (Biotek Cytation™ 3 Automated Fluorescence Microscope) (Agilent Technologies Inc., Santa Clara, CA, USA) plotted on a bar chart to evaluate the percentage of cell viability.

#### 4.4.1. Anti-Adhesion Assay

Vero cell lines at a density of 1 × 10^5^ were cultured in DMEM media supplemented with 10% FBS (Gibco, Grand Island, NY, USA) in 96-tissue culture plate (Corning Costar, Milano, Italy) at 37 °C in an atmosphere containing 5% CO_2_ for 24 h to all cells to attach. After 24 h, the media was removed, and 300 µL of *S. typhimurium* cells at an OD of 0.3 were added to the growing Vero cells. This was immediately followed with capsaicin, capsaicin extract, or media control supplemented DMEM media only. Infection was allowed for 30 min following a similar procedure by Manini et al., (2015) [28]. Final concentrations of capsaicin or capsaicin extract in the culture media were 1000 µg/mL, 100 µg/mL, 10 µg/mL, 1 µg/mL, 0.1 µg/mL, 0.01 µg/mL, and 0.001 µg/mL. Prior to infection of the Vero cells, *S. typhimurium* was freshly grown to an OD of 0.5, harvested, and pelleted via centrifugation. Bacteria pellets were washed thrice with 1X PBS and resuspended in DMEM media to an OD of 0.3.

To investigate the ability of *S. typhimurium* to attach to the monolayer Vero cells, the wells containing the infected Vero cells, or the controls were washed 3 times with 1X PBS and lysed with chilled distilled water. Then 300 µL of the lysed cells was spread to LB agar plates and incubated overnight at 37 °C. The colony forming units (CFU) were counted to evaluate the total adherent bacteria in each 300 µL of the lysate.

#### 4.4.2. Anti-Invasion Assay

The anti-invasion capacity of capsaicin or capsaicin extract was evaluated by counting the viable bacteria that survived the antibiotic insults due to their intracellular presence inside the host cell’s cytoplasm. To investigate the intracellular presence of *S. typhimurium* in infected Vero cells, infected monolayers were washed 3 times with 1X PBS as previously demonstrated by Manini et al., (2015) [28]. This was followed with the administration of penicillin 1 mL of bactericidal concentrations of penicillin (5 µg/mL) and gentamicin (100 µg/mL), and the samples were allowed to sit for 2 h at 37 °C in 5% CO_2_. The treated monolayers were washed and lysed with chilled distilled water, then viable intracellular bacteria were assayed by plating 300 µL of the lysate on an LB agar plate and cultured overnight. Each assay was repeated thrice.

#### 4.4.3. Statistical Analyses

For all statistical data, values were derived from multiple measurements (from replicates of 3 experiments) and averaged. Differences between groups were assessed with a paired Student’s *t*-test using OriginPro software (OriginLab Corporation, North Hampton, MA, USA). Values are reported as mean ± SEM. *p*-values ≤ 0.05 were considered statistically significant.

## 5. Conclusions

In conclusion, we investigated the effect of pure capsaicin and capsaicin extract from *C. chinense* on *S. typhimurium* and demonstrated that pure capsaicin and capsaicin extract showed potent antibacterial activity against this *Salmonella typhimurium*. At higher concentrations of capsaicin extract, we observed a reduction in bacterial cell growth, and this reduction in the growth of the bacteria was comparable to one of the effective antibiotics, ampicillin. Both pure capsaicin and capsaicin extract at lower concentrations were observed to have minimum effect on host cells (Vero cells) viability in vitro. Using biochemical assays, we observed that the mechanism of action of both pure capsaicin and capsaicin extract on *S. typhimurium* was because of bacterial membrane disruption. Furthermore, the observed capsaicin effect on *S. typhimurium* inhibition might also be due in part to other factors such as reactive oxygen species production, membranes leaks that disrupts bacterial energy biosynthesis, and the intracellular influx of ions that can potentially disrupt bacterial cellular processes or capsaicin involvement in bacterial lipid oxidation.

## Data Availability

Not applicable.

## Supplementary Materials

The following are available online at https://www.mdpi.com/article/10.3390/antibiotics11050666/s1, Figure S1: Immunofluorescent images of *S. typhimurium* growing on culture media pretreated with 100 µg/mL of pure capsaicin or capsaicin extract or ampicillin. Figure S2: Immunofluorescent images of *S. typhimurium* growing on culture media pretreated with 10 µg/mL of pure capsaicin or capsaicin extract or ampicillin. Figure S3: Immunofluorescent images of *S. typhimurium* growing on culture media pretreated with 2µg/mL of pure capsaicin or capsaicin extract or ampicillin. Figure S4: The effect of capsaicin or capsaicin extract on Vero cell viability at varying doses. Figure S5: Bar charts illustrating the average CFUs of *S. typhimurium* in bacteria adhesion and invasion assays. Figure S6: *S. typhimurium* membrane integrity assessment via agarose gel electrophoresis.

## References

[1] Voetsch A.C., Van Gilder T.J., Angulo F.J., Farley M.M., Shallow S., Marcus R., Cieslak P.R., Deneen V.C., Tauxe R.V., Emerging Infections Program FoodNet Working Group (2004). FoodNet estimate of the burden of illness caused by nontyphoidal *Salmonella* infections in the United States. Clin. Infect. Dis..

[2] Swaminathan B., Barrett T.J., Fields P. (2006). Surveillance for human *Salmonella* infections in the United States. J. AOAC Int..

[3] Leekitcharoenphon P., Hendriksen R.S., Le Hello S., Weill F.X., Baggesen D.L., Jun S.R., Ussery D.W., Lund O., Crook D.W., Wilson D.J. (2016). Global genomic epidemiology of *Salmonella enterica* serovar Typhimurium DT104. Appl. Environ. Microbiol..

[4] Finstad S., O’Bryan C.A., Marcy J.A., Crandall P.G., Ricke S.C. (2012). *Salmonella* and broiler processing in the United States: Relationship to foodborne salmonellosis. Food Res. Int..

[5] Olsen S.J., DeBess E.E., McGivern T.E., Marano N., Eby T., Mauvais S., Balan V.K., Zirnstein G., Cieslak P.R., Angulo F.J. (2001). A nosocomial outbreak of fluoroquinolone-resistant *Salmonella* infection. N. Engl. J. Med..

[6] Foley S.L., Johnson T.J., Ricke S.C., Nayak R., Danzeisen J. (2013). *Salmonella* pathogenicity and host adaptation in chicken-associated serovars. Microbiol. Mol. Biol. Rev..

[7] Maurelli A.T. (2007). Black holes, antivirulence genes, and gene inactivation in the evolution of bacterial pathogens. FEMS Microbiol. Lett..

[8] Butaye P., Michael G.B., Schwarz S., Barrett T.J., Brisabois A., White D.G. (2006). The clonal spread of multidrug-resistant non-typhi *Salmonella* serotypes. Microbes Infect..

[9] Giles W.P., Benson A.K., Olson M.E., Hutkins R.W., Whichard J.M., Winokur P.L., Fey P.D. (2004). DNA sequence analysis of regions surrounding bla CMY-2 from multiple *Salmonella* plasmid backbones. Antimicrob. Agents Chemother..

[10] Majid R., Demla V., Mohammed A.O.M., Friedman E.R., Kee P., Schmitt K. (2018). *Salmonella enteritidis* concurrent spinal epidural abscess, urinary tract infection and endocarditis in an immunocompetent host: Case report and a review of the literature. J. Trop. Dis..

[11] Hakim S., Davila F., Amin M., Hader I., Cappell M.S. (2018). Infectious aortitis: A life-threatening endovascular complication of nontyphoidal salmonella bacteremia. Case Rep. Med..

[12] Scallan E., Griffin P.M., Angulo F.J., Tauxe R.V., Hoekstra R.M. (2011). Foodborne illness acquired in the United States—Unspecified agents. Emerg. Infect. Dis..

[13] Martin L.J., Fyfe M., Doré K., Buxton J.A., Pollari F., Henry B., Middleton D., Ahmed R., Jamieson F., Ciebin B. (2004). Increased burden of illness associated with antimicrobial-resistant *Salmonella enterica* serotype Typhimurium infections. J. Infect. Dis..

[14] Helms M., Simonsen J., Mølbak K. (2004). Quinolone resistance is associated with increased risk of invasive illness and death in *Salmonella* Typhimurium infection. J. Infect. Dis..

[15] Hussain A., Satti L., Hanif F., Zehra N.M., Nadeem S., Bangash T.M., Peter A. (2019). Typhoidal *Salmonella* strains in Pakistan: An impending threat of extensively drug-resistant *Salmonella* Typhi. Eur. J. Clin. Microbiol. Infect. Dis..

[16] Brown A.C., Grass J.E., Richardson L.C., Nisler A.L., Bicknese A.S., Gould L.H. (2017). Antimicrobial resistance in *Salmonella* that caused foodborne disease outbreaks: United States, 2003–2012. Epidemiol. Infect..

[17] Chatham-Stephens K., Medalla F., Hughes M., Appiah G.D., Aubert R.D., Caidi H., Angelo K.M., Walker A.T., Hatley N., Masani S. (2019). Emergence of extensively drug-resistant *Salmonella* Typhi infections among travelers to or from Pakistan—United States, 2016–2018. Morb. Mortal. Wkly. Rep..

[18] Ahmed A.M., Shimamoto T., Shimamoto T. (2014). Characterization of integrons and resistance genes in multidrug-resistant *Salmonella enterica* isolated from meat and dairy products in Egypt. Int. J. Food Microbiol..

[19] Varma J.K., Mølbak K., Barrett T.J., Beebe J.L., Jones T.F., Rabatsky-Ehr T., Smith K.E., Vugia D.J., Chang H.G.H., Angulo F.J. (2005). Antimicrobial-resistant nontyphoidal *Salmonella* is associated with excess bloodstream infections and hospitalizations. J. Infect. Dis..

[20] Gildea L., Ayariga J.A., Ajayi O.S., Xu J., Villafane R., Samuel-Foo M. (2022). Cannabis sativa CBD Extract Shows Promising Antibacterial Activity against *Salmonella typhimurium* and *S. newington*. Molecules.

[21] Haruna A., Yahaya S.M. (2021). Recent Advances in the Chemistry of Bioactive Compounds from Plants and Soil Microbes: A Review. Chem. Afr..

[22] Abugri D.A., Ayariga J.A., Tiimob B.J., Yedjou C.G., Mrema F., Witola W.H. (2019). Medicinal mushrooms as novel sources for new antiparasitic drug development. Medicinal Mushrooms.

[23] Papoiu A.D., Yosipovitch G. (2010). Topical capsaicin. The fire of a ‘hot’medicine is reignited. Expert Opin. Pharmacother..

[24] Reyes-Escogido M.D.L., Gonzalez-Mondragon E.G., Vazquez-Tzompantzi E. (2011). Chemical and pharmacological aspects of capsaicin. Molecules.

[25] Haanpää M., Treede R.D. (2012). Capsaicin for neuropathic pain: Linking traditional medicine and molecular biology. Eur. Neurol..

[26] Narang N., Jiraungkoorskul W., Jamrus P. (2017). Current understanding of antiobesity property of capsaicin. Pharmacogn. Rev..

[27] Omolo M.A., Wong Z.Z., Borh W.G., Hedblom G.A., Dev K., Baumler D.J. (2018). Comparative analysis of capsaicin in twenty-nine varieties of unexplored Capsicum and its antimicrobial activity against bacterial and fungal pathogens. J. Med. Plants Res..

[28] Marini E., Magi G., Mingoia M., Pugnaloni A., Facinelli B. (2015). Antimicrobial and anti-virulence activity of capsaicin against erythromycin-resistant, cell-invasive group A streptococci. Front. Microbiol..

[29] Anogianaki A., Negrev N.N., Shaik Y.B., Castellani M.L., Frydas S., Vecchiet J., Tete S., Salini V., De Amicis D., De Lutiis M.A. (2007). Capsaicin: An irritant anti-inflammatory compound. J. Biol. Regul. Homeost. Agents.

[30] Cao S., Chen H., Xiang S., Hong J., Weng L., Zhu H., Liu Q. (2015). Anti-cancer effects and mechanisms of capsaicin in chili peppers. Am. J. Plant Sci..

[31] Ayariga J., Abugri D.A., Griffin G.D. (2021). Capsaicin and Dihydrocapsaicin Extracted from *Capsicum chinense* Decrease Cell Viability of Neuroblastoma SH-SY5Y Cells in Vitro. Preprints.

[32] Sun F., Xiong S., Zhu Z. (2016). Dietary capsaicin protects cardiometabolic organs from dysfunction. Nutrients.

[33] Sharma S.K., Vij A.S., Sharma M. (2013). Mechanisms and clinical uses of capsaicin. Eur. J. Pharmacol..

[34] Lejeune M.P., Kovacs E.M., Westerterp-Plantenga M.S. (2003). Effect of capsaicin on substrate oxidation and weight maintenance after modest body-weight loss in human subjects. Br. J. Nutr..

[35] Di Cara F., Andreoletti P., Trompier D., Vejux A., Bülow M.H., Sellin J., Lizard G., Cherkaoui-Malki M., Savary S. (2019). Peroxisomes in immune response and inflammation. Int. J. Mol. Sci..

[36] Qiu J., Niu X., Wang J., Xing Y., Leng B., Dong J., Li H., Luo M., Zhang Y., Dai X. (2012). Capsaicin protects mice from community-associated methicillin-resistant *Staphylococcus aureus* pneumonia. PLoS ONE.

[37] Chatterjee S., Asakura M., Chowdhury N., Neogi S.B., Sugimoto N., Haldar S., Awasthi S.P., Hinenoya A., Aoki S., Yamasaki S. (2010). Capsaicin, a potential inhibitor of cholera toxin production in Vibrio cholerae. FEMS Microbiol. Lett..

[38] Das J., Deka M., Gogoi K. (2018). Antimicrobial Activity of Chilli Extracts (*Capsicum chinense*) Against Food Borne Pathogens *Escherichia coli* and *Staphylococcus aureus*. Int. J. Res. Anal. Rev. (IJRAR).

[39] Zeyrek F.Y., Oguz E. (2005). In vitro activity of capsaicin against *Helicobacter pylori*. Ann. Microbiol..

[40] Kar D., Bandyopadhyay S., Dimri U., Mondal D.B., Nanda P.K., Das A.K., Batabyal S., Dandapat P., Bandyopadhyay S. (2016). Antibacterial effect of silver nanoparticles and capsaicin against MDR-ESBL producing *Escherichia coli*: An in vitro study. Asian Pac. J. Trop. Dis..

[41] Helms M., Ethelberg S., Mølbak K. (2005). DT104 Study Group International *Salmonella typhimurium* DT104 infections, 1992–2001. Emerg. Infect. Dis..

[42] Herikstad H., Motarjemi Y., Tauxe R. (2002). *Salmonella* surveillance: A global survey of public health serotyping. Epidemiol. Infect..

[43] Oyedemi B.O., Kotsia E.M., Stapleton P.D., Gibbons S. (2019). Capsaicin and gingerol analogues inhibit the growth of efflux-multidrug resistant bacteria and R-plasmids conjugal transfer. J. Ethnopharmacol..

[44] Chatterjee S.N., Chaudhuri K. (2012). Gram-negative bacteria: The cell membranes. Outer Membrane Vesicles of Bacteria.

[45] Sharma N., Phan H.T., Yoda T., Shimokawa N., Vestergaard M.D.C., Takagi M. (2019). Effects of capsaicin on biomimetic membranes. Biomimetics.

[46] Magi G., Marini E., Facinelli B. (2015). Antimicrobial activity of essential oils and carvacrol, and synergy of carvacrol and erythromycin, against clinical, erythromycin-resistant Group A Streptococci. Front. Microbiol..

[47] Omolo M.A., Wong Z.Z., Mergen K., Hastings J.C., Le N.C., Reil H.A., Case K.A., Baumler D.J. (2014). Antimicrobial properties of chili peppers. J. Infect. Dis. Ther..

[48] National Center for Biotechnology Information (2022) PubChem Compound Summary for CID 2762649. 2014, Ethidium Monoazide Bromide. https://pubchem.ncbi.nlm.nih.gov/compound/Ethidium-monoazide-bromide.

[49] Hayman M., Kam P.C. (2008). Capsaicin: A review of its pharmacology and clinical applications. Curr. Anaesth. Crit. Care.

[50] Lundbaek J.A., Birn P., Tape S.E., Toombes G.E., Søgaard R., Koeppe R.E., Gruner S.M., Hansen A.J., Andersen O.S. (2005). Capsaicin regulates voltage-dependent sodium channels by altering lipid bilayer elasticity. Mol. Pharmacol..

