# Supplementary material for: Capsaicin Potently Blocks Salmonella typhimurium Invasion of Vero Cells
Source: Antibiotics (Basel). 2022 May 16;11(5):666. doi: 10.3390/antibiotics11050666 (PMC9137802; doi:10.3390/antibiotics11050666)
Supplement: Supplementary file 1 [file antibiotics-11-00666-s001.zip › antibiotics-1709065-supplementary.pdf]

Supplementary Figures

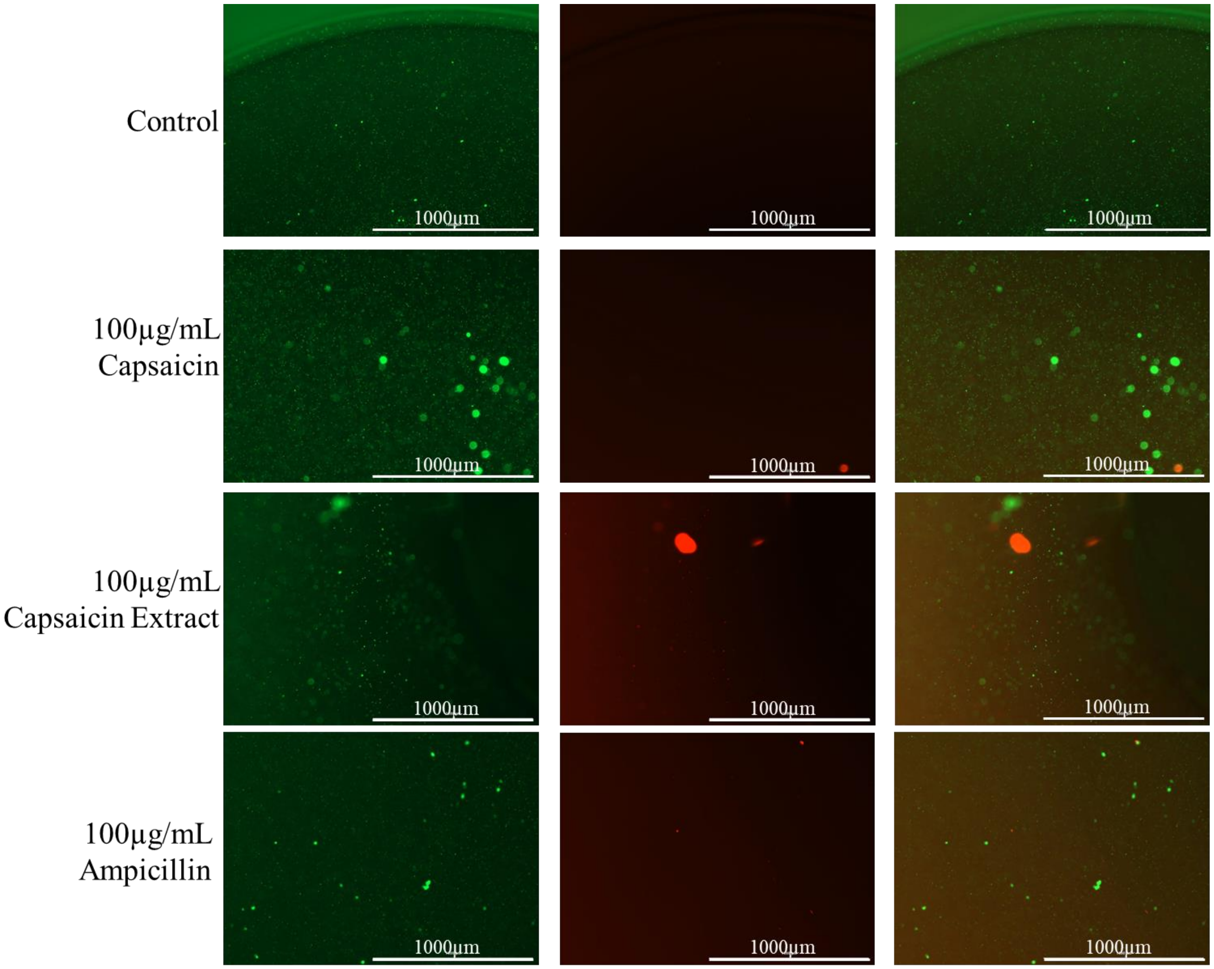

Figure S1. Immunofluorescent images of *S. typhimurium* growing on culture media pretreated with 100 µg/mL of pure capsaicin or capsaicin extract or ampicillin (30 min incubation in presence of capsaicin or capsaicin extract). Control received no treatment. Undamaged bacterial membrane shows green fluorescence, but those with damaged membranes shows red fluorescence.

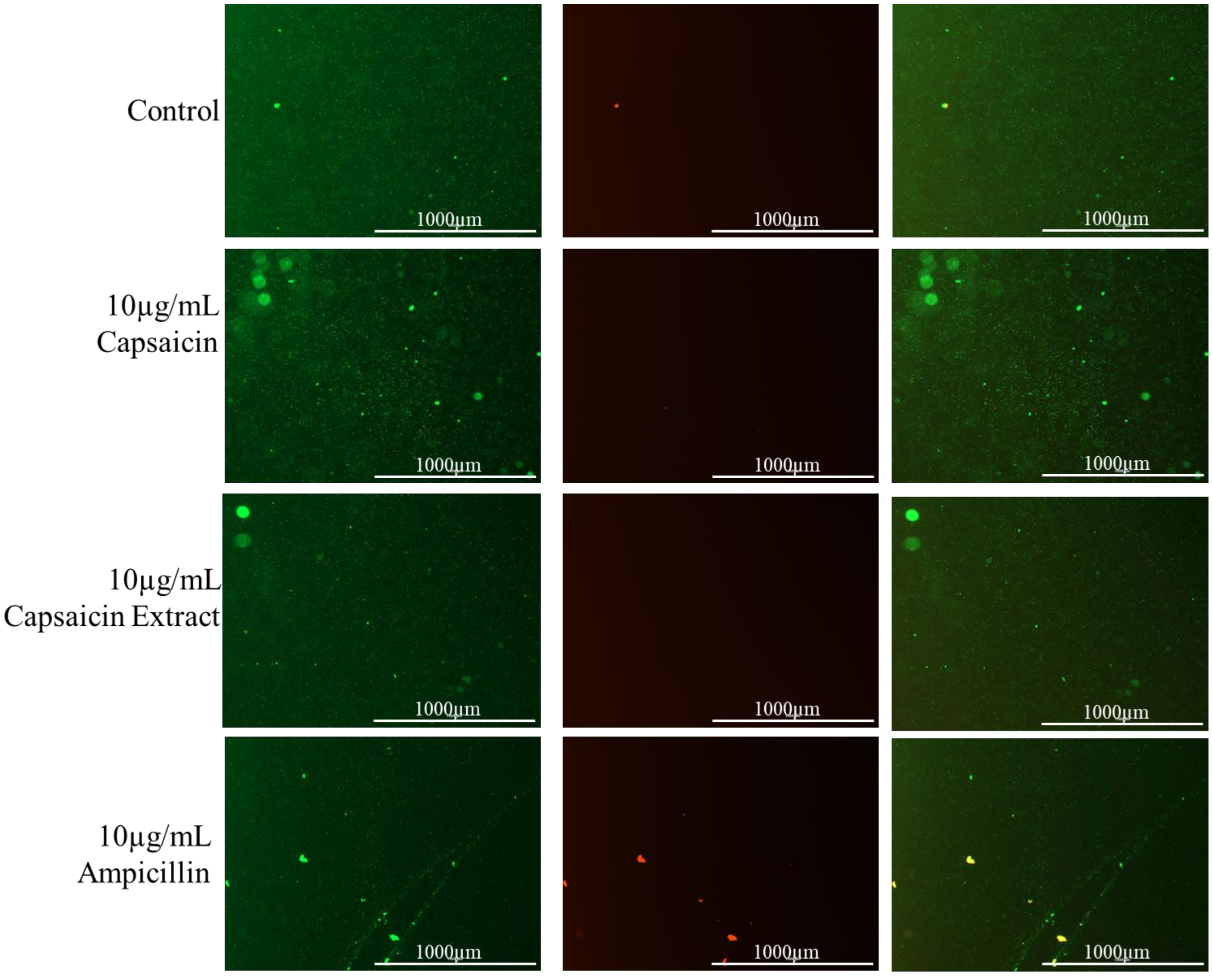

Figure S2. Immunofluorescent images of S. typhimurium growing on culture media pretreated with 10 µg/mL of pure capsaicin or capsaicin extract or ampicillin (30 min incubation in presence of capsaicin or capsaicin extract). Control received no treatment. Undamaged bacterial membrane shows green fluorescence, but those with damaged membranes shows red fluorescence.

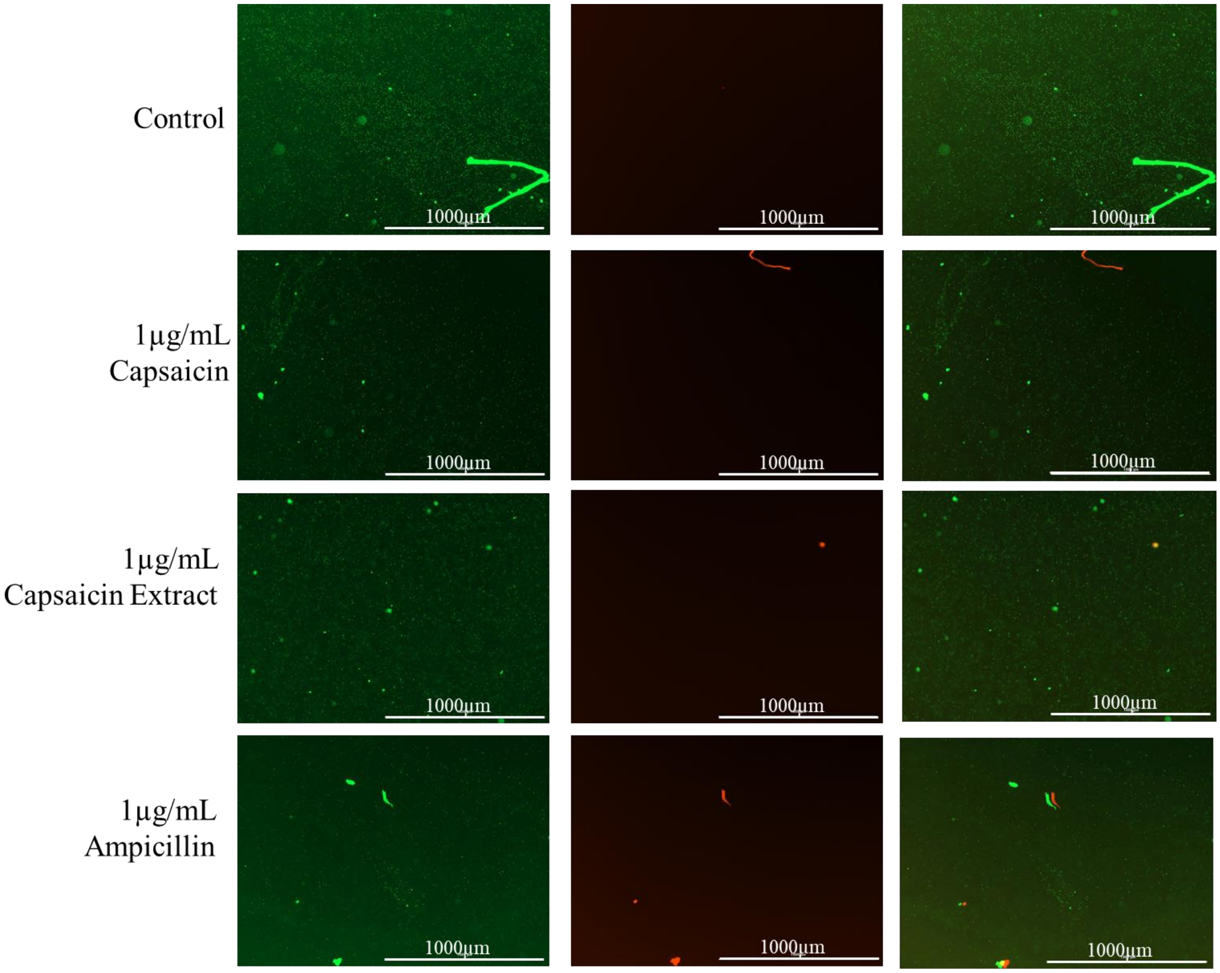

Figure S3. Immunofluorescent images of S. typhimurium growing on culture media pretreated with 2µg/mL of pure capsaicin or capsaicin extract or ampicillin (30 min incubation in presence of capsaicin or capsaicin extract). Control received no treatment. Undamaged bacterial membrane shows green fluorescence, but those with damaged membranes shows red fluorescence.

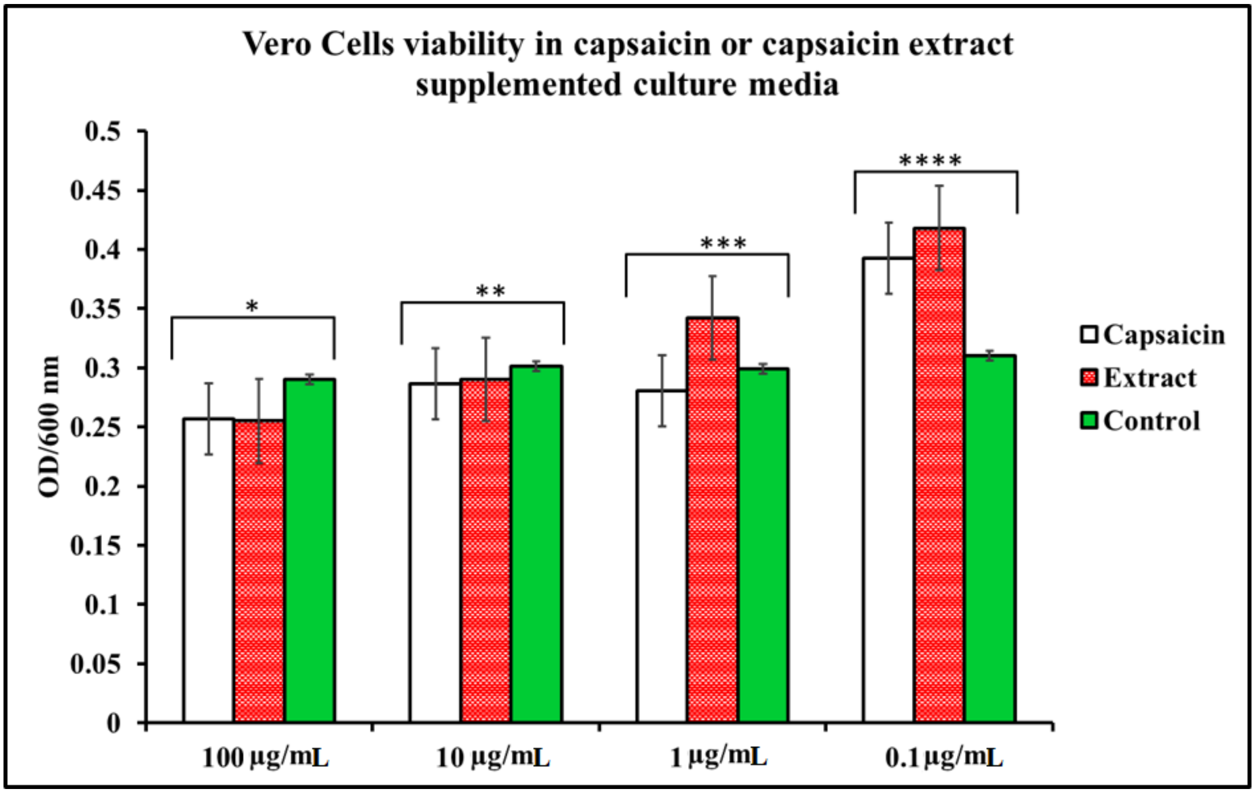

Figure S4. The effect of capsaicin or capsaicin extract on Vero cell viability at varying doses. *, **, *** and **** p-value ≤ 0.1; n = 3.

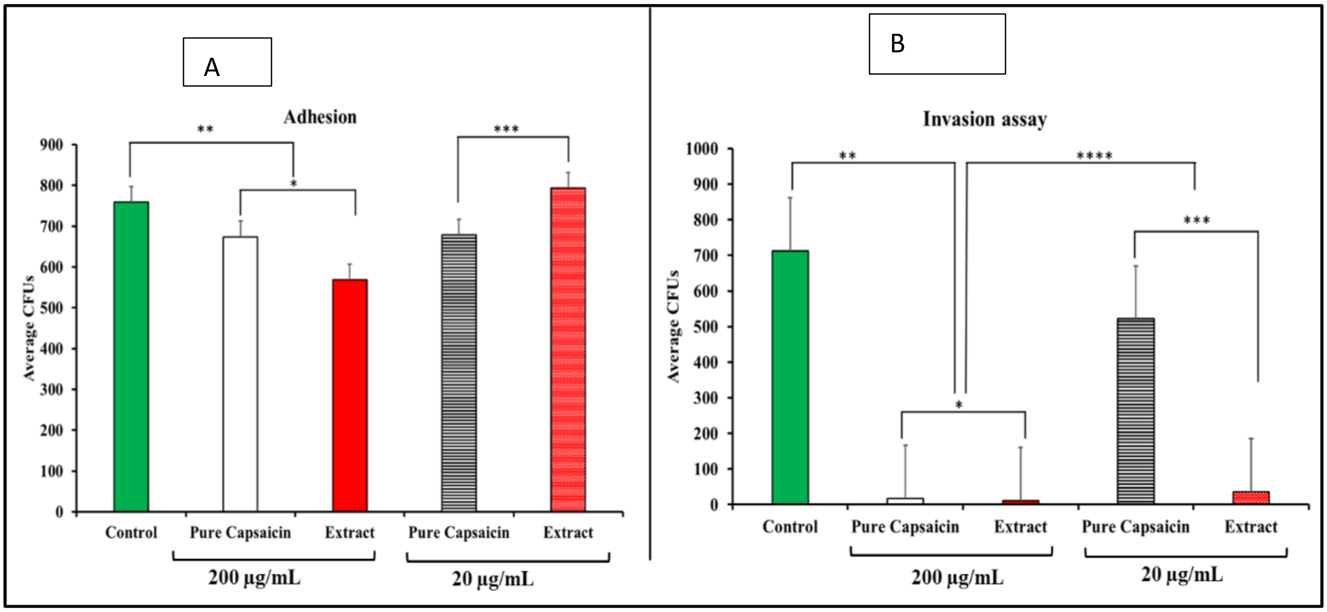

Figure S5. Bar charts illustrating the average CFUs of *S. typhimurium* in bacteria adhesion (**A**) and invasion assays (**B**). Monolayer Vero cells growing in media supplemented with capsaicin at varying concentrations were infected with *S. typhimurium* and incubated for 2 h to allow for bacterial cell invasion of Vero cells. Then the infected monolayer Vero cells were washed thrice with 1X PBS to remove bacterial cells in suspension and followed with antibiotic treatment for 2 h at 37 °C in 5% CO_2 to kill bacterial cells that adhered to Vero cells but did not internalize. Then antibiotics were washed off and Vero cells lysed with chilled distill water and plated on Agar plates overnight. The treated samples received capsaicin extract at 200 µg/mL or 20 µg/mL respectively, whereas the control received 1X PBS. For **(A)**; * and ** p-value ≤ 0.1, *** p-value ≤ 0.05; n = 3. For **(B)**; * p-value ≤ 0.361, **, *** and ****p-value ≤ 0.005; n = 3.

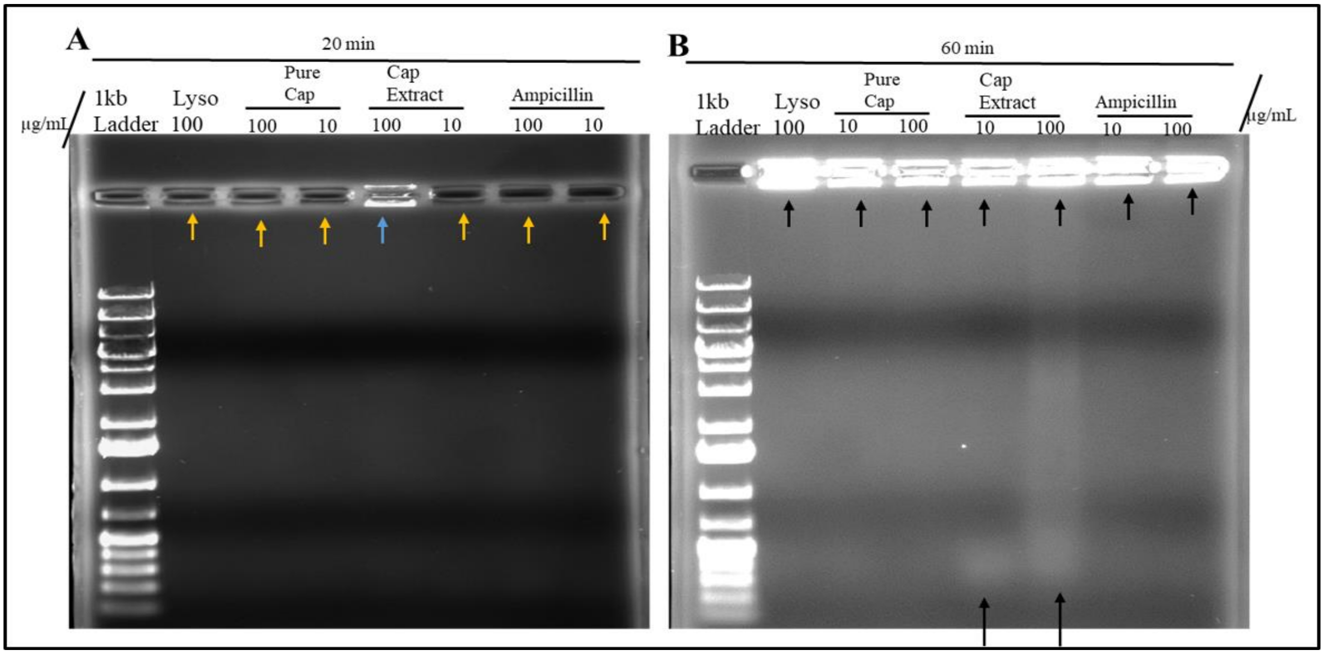

Figure S6. *S. typhimurium* membrane integrity assessment via agarose gel electrophoresis. (**A**) Bacterial cells were incubated with drugs and positive controls at 10 and 100 µg/mL for 20 min. (**B**) Bacterial cells were incubated in the concentrations mentioned in (**A**) above for 60 min. Gels were run at 100 volts for 60 min. Trail of blurry band can be observed in (**B**) as shown by the black arrows at capsaicin extract (Cap extract) lane 100 µg/mL. A fainter band can be observed at the 10 µg/mL lane too of capsaicin extract. In (**A**) no blurry band trail can be seen. Pure cap = pure capsaicin.
